# Posterior fossa arachnoid cyst causing torticollis and gastro-oesophageal reflux in an infant

**DOI:** 10.1007/s00381-018-3917-4

**Published:** 2018-07-30

**Authors:** John Hanrahan, Joseph Frantzias, Jose P. Lavrador, Istvan Bodi, Bassel Zebian

**Affiliations:** 1 Faculty of Life Sciences and Medicine, King’s College London, London, UK; 2 Department of Neurosurgery, King’s College Hospital, London, UK; 3 Department of Clinical Neuropathology, King’s College Hospital, London, UK

**Keywords:** Arachnoid cyst, Torticollis, GOR, Hydrocephalus, Endoscopy, Posterior fossa

## Abstract

**Introduction:**

Arachnoid cysts (ACs) account for a small proportion of all intracranial lesions. They are often incidental but can become symptomatic and even cause a threat to life. Symptoms are usually due to direct compression of neural elements and/or raised intracranial pressure.

**Case report:**

We report the case of an infant with an enlarging posterior fossa arachnoid cyst (PFAC) causing torticollis and gastro-oesophageal reflux (GOR), the combination of which had been previously unreported in this context. Endoscopic fenestration and cyst decompression were followed by complete resolution of the symptoms. We discuss the possible mechanisms of torticollis and GOR in this context.

## Introduction

Arachnoid cysts (ACs) are congenital lesions [1–4] that account for approximately 1% of all intracranial lesions. They most commonly occur in the middle and posterior cranial fossae [5, 6], and are usually incidental findings on imaging reflecting their typically benign nature [7]. Few ACs present with symptoms and may require neurosurgical intervention. We report a rare presentation of posterior fossa arachnoid cyst (PFAC) with torticollis and gastro-oesophageal reflux (GOR) in a child, which resolved following endoscopic cyst fenestration.

## Case report

**Fig. 1:**
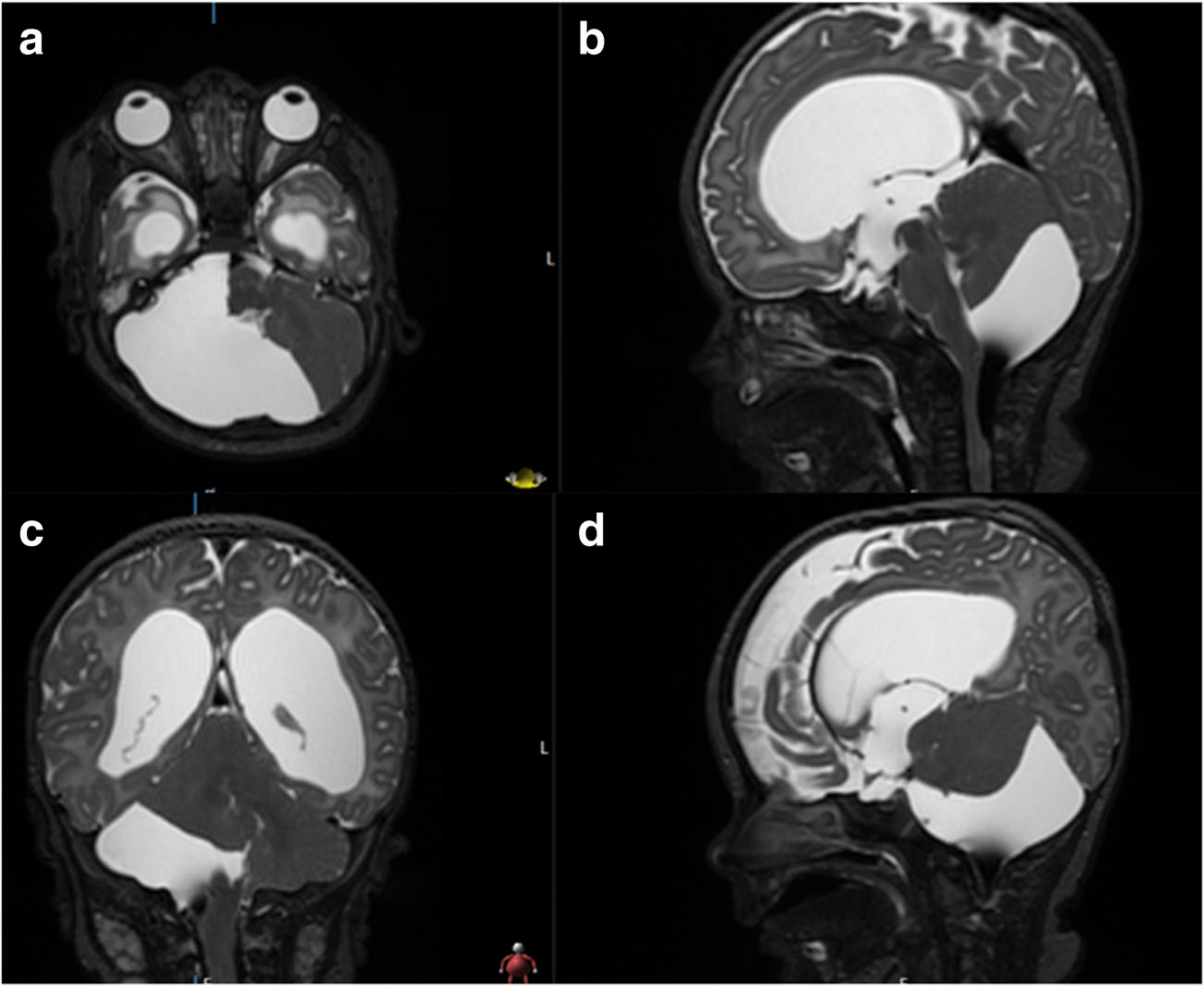
T2-weighted MRI demonstrating hydrocephalus and a posterior fossa AC on initial presentation; axial **a**; midline sagittal **b**; coronal **c**; paramedian sagittal **d**

A 2-month-old male infant was referred to the emergency department with macrocephaly. He was born at term via a normal vaginal delivery. Antenatal screening was normal with no initial post-natal concerns. Two weeks prior to admission, the head circumference increased significantly, and he started to have difficulty feeding with severe GOR. On examination, the anterior fontanelle was bulging and tense with prominent scalp veins. Urgent CT followed by MRI (Fig. 1) of the head demonstrated obstructive hydrocephalus due to a PFAC.

An endoscopic third ventriculostomy (ETV) was performed and a Rickham reservoir connected to an intraventricular catheter was inserted. The post-operative scan revealed decompression of the ventricular system and a stable PFAC. The infant was discharged home 3 days later.

**Fig. 2:**
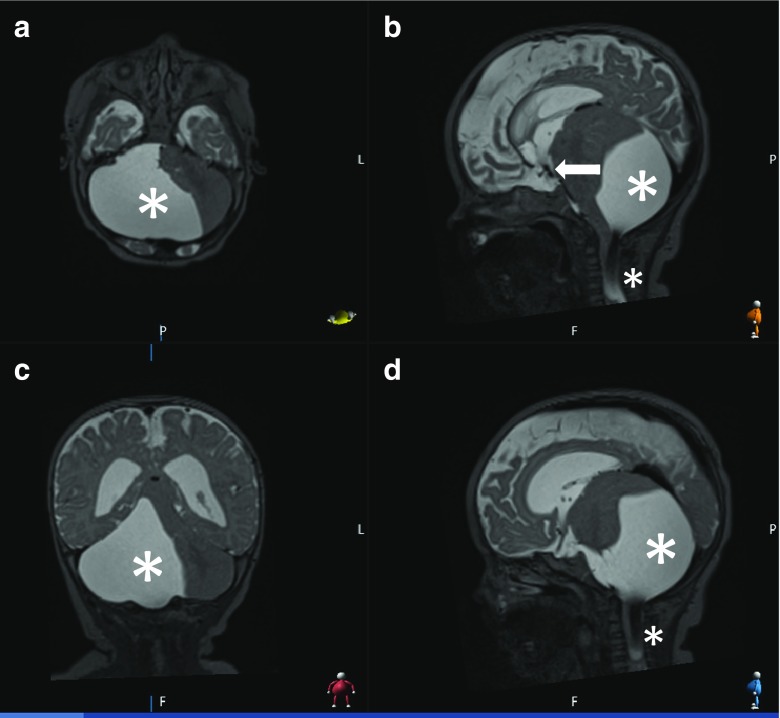
T2-weighted MRI demonstrating the increase in size of the AC with extension into the craniocervical junction (asterisks) with a functioning ventriculostomy (arrow); axial **a**; sagittal **b**; coronal **c**; paramedian sagittal **d**

In the following weeks, he developed torticollis (left lateral flexion) and GOR refractory to medical treatment. A repeat MRI revealed an increase in the size of the PFAC such that it was extending into the spinal canal through the craniocervical junction and causing significant mass effect on the brainstem. The previous ETV was still functioning (Fig. 2).

**Fig. 3:**
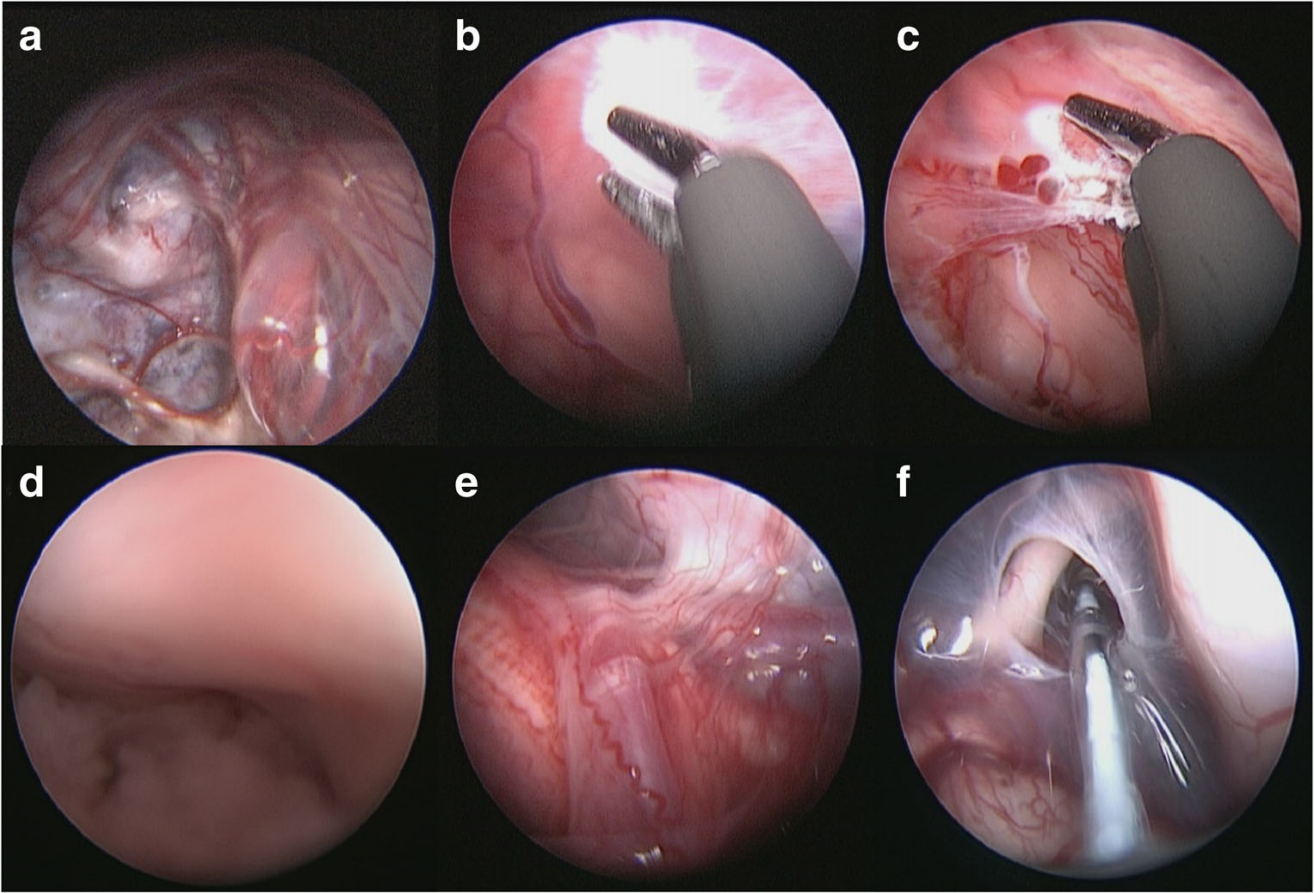
Intraoperative imaging demonstrating the inferior aspect of the cyst bulging up into the cavity **a**; cyst wall being coagulated **b**; choroid plexus lining cyst wall **c**; right foramen of Lushka **d**; lateral aspect of brainstem with the right lower cranial nerves and vertebral artery/PICA seen **e**; fenestration into pre-pontine cistern **f**

**Fig. 4:**
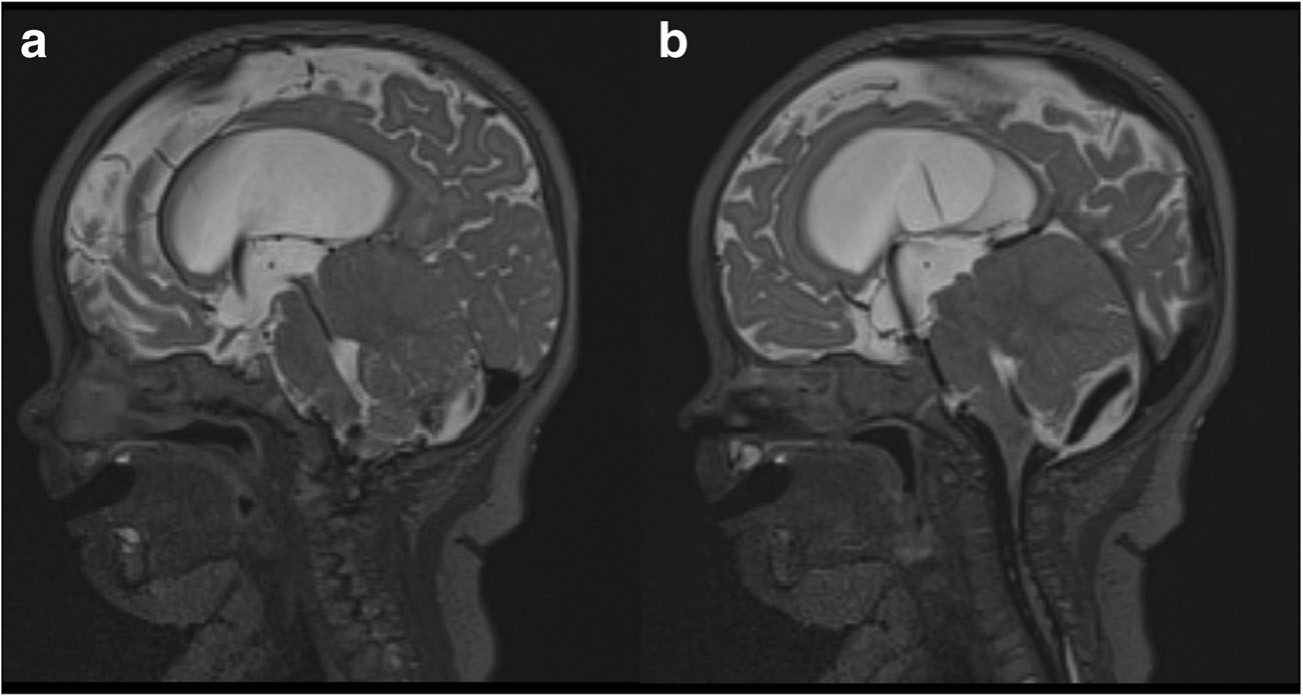
Postoperative MRI scan demonstrating reduction in size of posterior fossa AC after fenestration; sagittal view with flow through the aqueduct **a**; sagittal view with flow through ventriculostomy and outlets of the 4^th^ ventricle **b**

We proceeded with endoscopic cyst fenestration. The cyst was entered and its wall was coagulated in places (to reduce its size) then fenestrated. The fenestrations were into the craniocervical junction, the fourth ventricle, out through the right foramen of Luschka and into the pre-pontine cistern. Choroid plexus was seen within the cyst (Fig. 3c). Neuropathologically the cyst was consistent with an AC. The symptoms resolved post-operatively, with significant reduction in cyst size after fenestration (Fig. 4). The child was discharged home 4 days later.

He was readmitted with a CSF leak 1 week later. A CT scan demonstrated that the cyst was smaller and ruled out hydrocephalus. A trans-fontanelle tap revealed a raised white cell count with no organisms detected. He returned to surgery where the reservoir and intraventricular catheter were removed and replaced by an external ventricular drain (EVD). He received 14 days of intrathecal (IT) vancomycin and 16 days of intravenous meropenem and vancomycin. He made a good recovery. The EVD was removed and he was discharged home 20 days after surgery.

Despite reduction in the size of the cyst and a functioning ventriculostomy, the patient developed communicating hydrocephalus likely due to the infection. A left ventriculoperitoneal shunt was inserted after serial lumbar punctures. At 18-month follow-up he is fit and well, with no recurrence of symptoms.

## Discussion

There are four previous reports of PFACs presenting with torticollis [8–10] (Table 1). Zaher et al. reported the only other PFAC presenting with torticollis managed endoscopically [10] which achieved resolution of symptoms without complication. Similarly, we observed resolution of torticollis and GOR following fenestration.

**Table 1:** Existing reports of posterior fossa arachnoid cysts presenting with torticollis

Study	Presenting features	Age/gender	Treatment	Outcome
Per et al. 2014 [11]	Torticollis, macrocephaly	16-month-old male	Cystoperitoneal shunt	Significant improvement of torticollis with no reported complications
Zaher et al. 2015 [12]	Torticollis	Unable to elicit	Endoscopic fenestration	Significant improvement with resolution of torticollis
Fulkerson et al. 2011 [13]	Torticollis, enlarging occipitofrontal circumference; plagiocephaly	8-month-old male	Stereotactic placement of cyst-ventricle stent	Clinically stable with decrease in cyst size at 5-year follow-up. Slight delay in milestones
Tumturk et al. 2015 [14]	Torticollis, left eye esotropia	12-month-old female	Refused	Unknown
Current case	Torticollis, gastro-oesophageal reflux, macrocephaly	2-month-old male	Endoscopic fenestration	Resolution of symptoms with good neurological recovery at 18-month follow-up

The precise mechanism resulting in torticollis in the context of a PFAC is unclear. However, symptom resolution after cyst decompression implies a role of mass effect. Compression of structures such as the vermis and fastigial nucleus may explain this presentation due to their involvement in the control of head movement [15]. Other hypotheses include stretching and irritation of the dura of the ascending meningeal branches of C1–C3 nerves and the accessory nerve [16]. The atypical cervical extension of the cyst as demonstrated on the MRI (Fig. 2) may account for the unusual symptoms. This is supported by previous reports of tumours at the craniocervical junction presenting with torticollis [11–13].

We also observed GOR refractory to medical management resolve upon cyst fenestration. This association, to the best of our knowledge, has not been reported before. Vagal nerve impairment has been implicated in the pathogenesis of GOR [14], for example, with evidence of impaired reflux control in patients with vagus nerve injury subsequent to anti-reflux surgery [17]. As GOR developed after the cyst had increased in size, we suggest a similar process of vagal nerve impairment for the poor feeding and GOR.

A minority of PFACs require intervention [18]; at present, there is no consensus on the optimal approach. We initially treated the hydrocephalus using an ETV and did not address the cyst directly as AC growth is uncommon and PFAC fenestration is associated with higher operative risk [19–21]. Subsequent cyst growth resulting in torticollis and GOR despite a functioning ETV in our patient required further surgical intervention and justified the higher risk associated with cyst fenestration. The endoscopic approach for cyst fenestration has been reported to cause less surgical trauma and fewer complications than microsurgery [22, 23], and has been favoured over shunting due to lower levels of recurrence and complications [18]. Ultimately, a shunt was still required to treat the communicating hydrocephalus that developed.

## Conclusions

We report an unusual, and to the best of our knowledge, unique presentation with torticollis and GOR secondary to a PFAC in an infant which resolved upon fenestration. Whilst the exact mechanism causing such symptoms is unclear, resolution of mass effect treated the symptomatology and allowed normal development. This is the second report of a PFAC presenting with torticollis to be managed endoscopically and the first report of GOR in this context.

## References

[1] Cincu R, Agrawal A, Eiras J (2007). Intracranial arachnoid cysts: current concepts and treatment alternatives. Clin Neurol Neurosurg.

[2] Oberbauer RW, Haase J, Pucher R (1992). Arachnoid cysts in children: a European co-operative study. Childs Nerv Syst.

[3] Van Tassel P, Curé JK (1995). Nonneoplastic intracranial cysts and cystic lesions. Semin Ultrasound CT MR.

[4] FOWLER FD (1956). Atresia of the foramina of Luschka and Magendie. AMA J Dis Child.

[5] Galassi E, Tognetti F, Frank F, Fagioli L, Nasi MT, Gaist G (1985). Infratentorial arachnoid cysts. J Neurosurg.

[6] Al-Holou WN, Yew AY, Boomsaad ZE (2010). Prevalence and natural history of arachnoid cysts in children. J Neurosurg Pediatr.

[7] Seizeur R, Forlodou P, Coustans M, Dam-Hieu P (2007). Spontaneous resolution of arachnoid cysts: review and features of an unusual case. Acta Neurochir.

[8] Per H, Canpolat M, Tümtürk A, Gumuş H, Gokoglu A, Yikilmaz A, Özmen S, Kaçar Bayram A, Poyrazoğlu HG, Kumandas S, Kurtsoy A (2014). Different etiologies of acquired torticollis in childhood. Childs Nerv Syst.

[9] Fulkerson DH, Vogel TD, Baker AA, Patel NB, Ackerman LL, Smith JL, Boaz JC (2011). Cyst-ventricle stent as primary or salvage treatment for posterior fossa arachnoid cysts. J Neurosurg Pediatr.

[10] Zaher A, Nabeeh M, Gomaa M (2015). Endoscopic management of posterior fossa arachnoid cysts. Egypt J Neurosurg.

[11] Constantini S, Houten J, Miller DC, Freed D, Ozek MM, Rorke LB, Allen JC, Epstein FJ (1996). Intramedullary spinal cord tumors in children under the age of 3 years. J Neurosurg.

[12] Giuffrè R, Di Lorenzo N, Fortuna (1981) A Cervical tumors of infancy and childhood. J Neurosurg Sci 25(3-4):259–264 7346619

[13] Cruysberg JR, Draaijer RW, Snijders-Bosman PW (1998). Two children with a rare etiology of torticollis: primitive neuro-ectodermal tumor and Grisel’s syndrome. Ned Tijdschr Geneeskd.

[14] Ogilvie AL, James PD, Atkinson M (1985). Impairment of vagal function in reflux oesophagitis. Q J Med.

[15] Kumandaş S, Per H, Gümüş H, Tucer B, Yikilmaz A, Kontaş O, Coşkun A, Kurtsoy A (2006). Torticollis secondary to posterior fossa and cervical spinal cord tumors: report of five cases and literature review. Neurosurg Rev.

[16] Tumturk A, Kaya Ozcora G, Kacar Bayram A, Kabaklioglu M, Doganay S, Canpolat M, Gumus H, Kumandas S, Unal E, Kurtsoy A, Per H (2015). Torticollis in children: an alert symptom not to be turned away. Childs Nerv Syst.

[17] van Rijn S, Rinsma NF, van Herwaarden-Lindeboom MYA, Ringers J, Gooszen HG, van Rijn PJJ, Veenendaal RA, Conchillo JM, Bouvy ND, Masclee AAM (2016). Effect of vagus nerve integrity on short and long-term efficacy of antireflux surgery. Am J Gastroenterol.

[18] Marin-Sanabria EA, Yamamoto H, Nagashima T, Kohmura E (2007). Evaluation of the management of arachnoid cyst of the posterior fossa in pediatric population: experience over 27 years. Childs Nerv Syst.

[19] Wester K, Hugdahl K (1995). Arachnoid cysts of the left temporal fossa: impaired preoperative cognition and postoperative improvement. J Neurol Neurosurg Psychiatry.

[20] Bristol RE, Albuquerque FC, McDougall C, Spetzler RF (2007). Arachnoid cysts: spontaneous resolution distinct from traumatic rupture. Case report. Neurosurg Focus.

[21] Lee JY, Kim JW, Phi JH, Kim SK, Cho BK, Wang KC (2012). Enlarging arachnoid cyst: a false alarm for infants. Childs Nerv Syst.

[22] Choi JU, Kim DS, Huh R (1999). Endoscopic approach to arachnoid cyst. Childs Nerv Syst.

[23] Gangemi M, Seneca V, Colella G, Cioffi V, Imperato A, Maiuri F (2011). Endoscopy versus microsurgical cyst excision and shunting for treating intracranial arachnoid cysts. J Neurosurg Pediatr.

